# Common perinatal mental disorders and post‐infancy child development in rural Ethiopia: A population‐based cohort study

**DOI:** 10.1111/tmi.13725

**Published:** 2022-02-08

**Authors:** Julia Alexandra Dunn, Girmay Medhin, Michael Dewey, Atalay Alem, Bogale Worku, Diana Paksarian, Charles R. Newton, Mark Tomlinson, Martin Prince, Charlotte Hanlon

**Affiliations:** ^1^ Health Service and Population Research Department, Institute of Psychiatry, Psychology and Neuroscience, King’s Colleges London Centre for Global Mental Health London UK; ^2^ Genetic Epidemiology Research Branch, Intramural Research Program National Institute of Mental Health Bethesda Maryland USA; ^3^ Aklilu‐Lemma Institute of Pathobiology Addis Ababa University Addis Ababa Ethiopia; ^4^ Department of Psychiatry, WHO Collaborating Centre for Mental Health Research and Capacity‐Building, School of Medicine, College of Health Sciences Addis Ababa University Addis Ababa Ethiopia; ^5^ Department of Paediatrics and Community Health Addis Ababa University Addis Ababa Ethiopia; ^6^ Department of Psychiatry University of Oxford Oxford UK; ^7^ Department of Global Health, Institute for Life Course Health Research Stellenbosch University Cape Town South Africa; ^8^ School of Nursing and Midwifery Queens University Belfast UK; ^9^ King’s College London King’s Global Health Institute London UK; ^10^ Centre for Innovative Drug Development and Therapeutic Trials for Africa (CDT‐Africa) College of Health Sciences Addis Ababa University Addis Ababa Ethiopia

**Keywords:** child development, child mental health, cohort study, postnatal depression, sub‐Saharan Africa

## Abstract

**Objective:**

To investigate whether maternal common mental disorders (CMD) in the postnatal period are prospectively associated with child development at 2.5 and 3.5 years in a rural low‐income African setting.

**Methods:**

This study was nested within the *C*‐*MaMiE* (Child outcomes in relation to Maternal Mental health **i**n **E**thiopia) population‐based cohort in Butajira, Ethiopia, and conducted from 2005 to 2006. The sample comprised of 496 women who had recently given birth to living, singleton babies with recorded birth weight measurements, who were 15 to 44 years of age, and residing in six rural sub‐districts. Postnatal CMD measurements were ascertained 2 months after delivery. Language, cognitive, and motor development were obtained from the child 2.5 and 3.5 years after birth using a locally adapted version of the Bayley Scales of Infant Development (3rd Ed). Maternal CMD symptoms were measured using a locally validated WHO Self‐Reporting Questionnaire. A linear mixed‐effects regression model was used to analyze the relationship between postnatal CMD and child development.

**Results:**

After adjusting for confounders, there was no evidence for an association between postnatal CMD and overall child development or the cognitive sub‐domain in the preschool period. There was no evidence of effect modification by levels of social support, socioeconomic status, stunting, or sex of the child.

**Conclusions:**

Previous studies from predominantly urban and peri‐urban settings in middle‐income countries have established a relationship between maternal CMD and child development, which contrasts with the findings from this study. The risk and protective factors for child development may differ in areas characterized by high social adversity and food insecurity. More studies are needed to investigate maternal CMD’s impact on child development in low‐resource and rural areas.

## INTRODUCTION

Almost no evidence exists on the impact of postnatal mental health on post‐infancy child development from rural community settings in sub‐Saharan Africa. Maternal mental health is now recognised as an important factor for optimal child health, growth, and development. Pregnancy and the first year after childbirth comprise a vulnerable period for many women, as changes in roles and responsibilities occur [1]. The postnatal period presents a critical period for the child, with exposure to adversity having enduring effects from infancy through adulthood [2].

The symptoms of common mental disorders (CMD), including depressive, anxiety, or somatic symptoms, have been increasingly reported in the postnatal period among women [3]. Researchers have reported estimates of CMD in the perinatal period higher in low‐ and middle‐income countries (LMIC) than in high‐income countries (HIC) [4, 5, 6]. In a meta‐analysis of 38 studies, the prevalence estimates were 18% for depressive and 14% for anxiety symptoms among African women in the postnatal period [7]. In another meta‐analysis of 13 studies from LMIC, the estimated prevalence for non‐psychotic maternal CMD was 15.6% during pregnancy and 19.8% in the postpartum period [4].

The effect of postnatal CMD symptoms on child development has been studied extensively in HIC, with an adverse impact observed from infancy [8], throughout childhood [2, 9], and persisting into adolescence [10, 11]. Women with symptoms of postnatal CMD may have a reduced capacity to practice childcare duties and miss cues for interactions with their children [12, 13]. The first three years of a child's life are a critical period for development, particularly cognitive development [14] and socioemotional development [2, 9], initiating trajectories with impacts on academic achievements [15], mental and physical health outcomes [16], and productivity in adulthood [11]. Postnatal depression, when combined with social adversity [11] and having male offspring [17], has shown consistent associations with poorer cognitive outcomes, including delayed achievement of developmental milestones and impaired learning abilities [10, 11].

Researchers in LMIC have increasingly investigated the connection between maternal mental health and child outcomes but have predominantly focused on children's health, growth, and survival [18]. No consistent associations between postnatal CMD and child development have been found [19], with associations observed in some [9, 20, 21] but not all studies [22, 23, 24]. Previous studies were also limited by their cross‐sectional [20, 25], or case‐control design [26], reliance on parental self‐report of child development [2, 9], and use of non‐validated measures [9, 20]. The studies with an observed relationship were primarily conducted in World Bank‐defined middle‐income countries (MIC), where the factors that influence child development may differ from low‐income countries [9, 19, 26]. In settings with high rates of poverty and stunting, cognitive development may be particularly impacted due to delayed brain maturation and chronic undernutrition [27]. There is, therefore, a need for prospective studies investigating the impact of postnatal CMD on child development in the post‐infancy period in community‐based settings in low‐income countries.

This research study follows up on the same population‐based cohort of women and infants in rural Ethiopia [24]. In previous research with this cohort, no relationship was found between antenatal or postnatal CMD symptoms and any infant development domain at 12 months [24]. This study will elucidate the long‐term and cumulative impacts of postnatal CMD beyond the period of infancy in toddlers. Children in the preschool period have an increased ability to communicate compared with infants [28]. Particular developmental milestones of toddlerhood, such as cognitive and linguistic development, may be affected by reduced engagement and responsiveness, often accompanying symptoms of maternal CMD [29].

In this study, we investigate the relationship between postnatal CMD and preschool child development in a rural African setting characterised by high levels of social adversity and food insecurity. We hypothesised that maternal CMD occurring in the postnatal period would have an independent long‐term adverse effect on child development and cognitive outcomes at 2.5 and 3.5 years. We explore whether stunting and home environment mediated, and socioeconomic status, social support, and sex moderated this relationship. In addition, we investigate whether postnatal CMD was associated with a reduced change in child development between 2.5 and 3.5 years.

## METHODS

The *C*‐*MaMiE* study (Child outcomes in relation to Maternal Mental health in Ethiopia) is a population‐based cohort study [30]. Participants were recruited and assessed in pregnancy and underwent repeated assessments with the index child. Measurements in this analysis were taken at birth and 2 months, 2.5 and 3.5 years after delivery.

### Study setting

The *C*‐*MaMiE* study was conducted in the Health and Demographic Surveillance Site (HDSS) in the Butajira area, Ethiopia. The Butajira HDSS is 130 kilometres south of the capital, Addis Ababa, and was established in 1986 as part of the Butajira Rural Health Programme [31]. At the time of the study, the HDSS population was 49,943, with 13,268 women of reproductive age [30]. One general hospital exists in Butajira town, and a second hospital is located 8 km outside of town. In addition, four primary health centres and seven health posts serve the HDSS population. Rural residents rely on a livelihood based on mixed farming of cash crops, mainly khat and chilli peppers, maize as the subsistence grain, and false bananas. Parts of the HDSS are food insecure because of a combination of overpopulation and intermittent drought [32].

Health‐centre‐based nurses and experienced project data collectors were trained for 10 days by the project co‐investigator (G.M.) to administer the Bayley III. The co‐investigator has a Master's degree in applied statistics and experience working with the Bayley Scale in Butajira and was supported by an Ethiopian consultant paediatrician (B.W.) and an Ethiopian psychiatrist (A.A.). The paediatrician took a prominent role in observing the administration of the complete Bayley Scales by trainees, giving feedback, and discussing the findings in detail with the trainees. The data collectors and local female high‐school graduates surveyed using the HOME scale and structured demographic questionnaires. Both nurses and C‐MaMiE data collectors administered the Bayley III with comparably high reliability (Chronbach's α > 0.7) in a previous validation study [33].

For the *C*‐*MaMiE* study, a sample of 1065 women was recruited from 1234 eligible women (86.3%) in the Butajira HDSS between July 2005 and February 2006 [30]. HDSS enumerators identified participants during their routine quarterly surveillance interviews. After giving verbal or written informed consent, the participants were interviewed by data collectors in their own homes. Women aged 15 to 49 years, able to speak Amharic, residing in the HDSS, and in their third trimester of pregnancy were eligible. Women with a known severe mental disorder, such as psychotic or bipolar disorder, or an emergency health condition during enrolment were excluded.

The cohort was restricted to women with singleton, living births for the analytic sample, with birth weight measured within 48 hours of delivery, from rural sub‐districts (*kebeles*), and who had maternal CMD symptoms assessed 2 months postnatally. At the time of recruitment into the study, around 90% of deliveries took place at home [34]. In six rural sub‐districts, a community worker was trained to measure birth weight within 48 h of birth in the woman's home [24].

### Outcome measure

Child development was measured with a composite of three sub‐scales (cognitive, motor, and language development) on the Bayley Scales of Infant Development, third edition (Bayley III). The Bayley III has been translated into Amharic and validated in Butajira with this cohort [33]. Items lacking cultural validity were adapted (e.g., pictures adapted for contextual relevance) or dropped (e.g., involving scissors or stairs). No time limit was imposed for the completion of items. Mokken scaling, a method based on non‐parametric item response theory, was used to create a hierarchical scale for the raw scores at both time points [35, 36].

### Primary exposure

Postnatal CMD was measured 2 months after birth, using the WHO 20‐item version of the Self‐Reporting Questionnaire (SRQ‐20) [37]. The SRQ‐20 functions as a screening tool that assesses the presence or absence of depressive, anxiety, and somatic symptoms in the previous month. The measure has been used in other Ethiopian studies [38, 39] and was validated with this cohort as a continuous measure [40].

### Confounders, effect modifiers, and mediators

**FIGURE 1:**
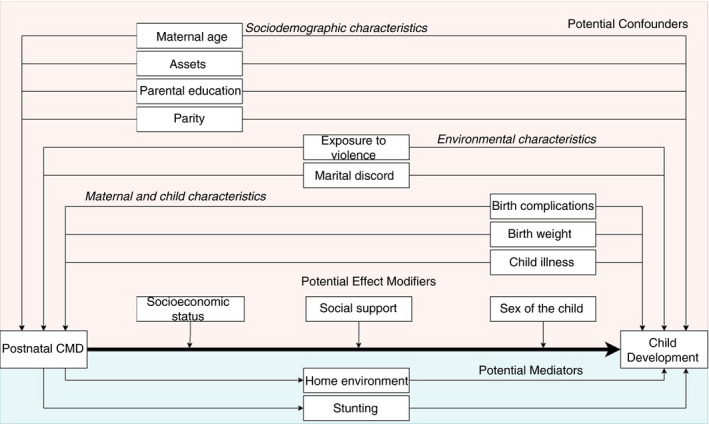
Conceptual framework for the association between postnatal maternal common mental disorders and child development

A conceptual framework was developed for this analysis based on previous theoretical models and literature on the risk factors for maternal CMD and child development (Figure 1) [21, 24, 41]. The selection of confounders, mediators, and confounders was theory‐driven. Factors were considered confounders if they were hypothesized to have a relationship with postnatal CMD and child development, affecting their relationship. In contrast, mediators were selected if they potentially explained the relationship between postnatal CMD and child development. Finally, effect modifiers were chosen if there was a hypothesis that the effect of postnatal CMD on child development varied across the levels of another variable. The measures were assessed at pregnancy, birth, and 2.5 and 3.5‐year timepoints (Figure S1).

During pregnancy, the following confounders were obtained through self‐report and included maternal age, parental education level, and parity [4]. Assets comprised ownership of 11 resources (e.g., land, house, crops). Socioeconomic status (SES) [4] was confirmed with Mokken scaling [38, 39] and included self‐report of hunger in the last month, indebtedness, lack of access to emergency resources, and perceived lower relative wealth. Marital discord [5] was summarised using Mokken scaling [24] and included self‐report of inadequate help from husband, relationship quality, frequency of quarrels, and perception of problematic alcohol consumption by the husband. Exposure to violence [4] assessed women's experience of physical violence since birth. Social support [11] comprised women's perception of the support received with housework and children.

The sex of the child [43] was obtained at birth. Obstetric complications [30] summed the responses to instrumental or operative delivery, duration of labour greater than 24 h, and bleeding or fever after delivery. Birth weight [44] was measured within 48 h of delivery using SECA 725 scales to an accuracy of 10 g [35].

Home environment, child growth or stunting, and child illness were measured 2.5 and 3.5 years after birth. Home environment [45] was measured using the original Home Observation for Measurement of Environment (HOME) scale [46]. The HOME measure of environmental stimulation was not formally validated for the setting. Because of difficulties with the contextual adaptation of the HOME, we relied on the sub‐scales based on observation of mother‐child interactions. The other sub‐scales were more challenging as they assessed aspects of a stimulating environment (i.e., number of books and time spent watching television) that were difficult to adapt for this low‐resource, rural setting. The instrument measures the amount and quality of stimulation and support provided to a child. Sub‐scales include a responsivity and an acceptance scale focused on the parent's attentiveness to the child and negative interactions. Height‐for‐Age Z scores [47] were calculated using WHO standard growth curves to define children as stunted at two Z‐scores below the median. Lower scores are indicative of higher levels of stunting in a child. Height‐for‐age has been argued to function as a better measure of cumulative undernutrition and more predictive of impaired child development [48]. A standard piece of medical equipment for height measurements, a stadiometer, was used to measure height with an adjustable headpiece. Child illness [11] was assessed through maternal recall for the presence of diarrhoea, fever, and severe illness episodes in the past 6 months.

SES [11], social support [11], stunting, and sex of the child [17], were conceptualised as potential effect modifiers, and stunting [9] and home environment [12] as potential mediators.

### Statistical analyses

The analysis was conducted using Stata Version 16. Participants’ characteristics with missing data on the primary outcome were compared with those remaining in the cohort, using Pearson chi‐squared tests, t‐tests, and Wilcoxon rank‐sum tests.

### Hypothesis‐driven analyses

The multivariable analysis of the association between postnatal maternal CMD symptoms and total and cognitive development outcomes was hypothesis‐driven. A mixed‐effects linear regression model with a random intercept was fitted. Model fit was tested using likelihood ratio tests after adding random slopes. An interaction with time was included to estimate the association between postnatal CMD and the change in child development between 2.5‐ and 3.5‐year time points.

All conceptualised confounders were added into the multivariable model sequentially, clustered by socio‐demographic, maternal and child, and environmental characteristics. The final model included all *a priori* confounders (Figure 1). Effect modification was investigated by including interaction terms for SES, sex of the child, stunting, and social support. Home environment and stunting were individually added to the final model to assess for exploratory evidence of mediation.

### Exploratory analyses

1. The relationship was investigated between preschool exposure to maternal CMD symptoms at three years and child development at 3.5 years using linear regression to determine the sensitivity of the maternal CMD measurement's time point.
2. A post‐hoc analysis was conducted to explore a threshold effect for postnatal CMD’s adverse impact on child development. Based on previous Ethiopian validation studies, we considered the SRQ‐20 a binary categorical exposure with a cut‐off score of ≥6 to indicate high CMD symptoms [40].

### Ethical considerations

The National Ethical Review Committee for Ethiopia and the Research Ethics Committee of King's College London in the U.K. approved the *C*‐*MaMiE* study. All participants gave informed consent. Literate women provided written consent, and non‐literate women indicated their consent with a thumbprint. Women received reimbursement for healthcare costs, and participants suffering from severe mental disorders, including psychotic or bipolar disorder, were referred to the local psychiatric unit and covered transportation costs. At baseline, women experiencing violence were directed to a local, community‐based non‐governmental organization for services.

## RESULTS

**FIGURE 2:**
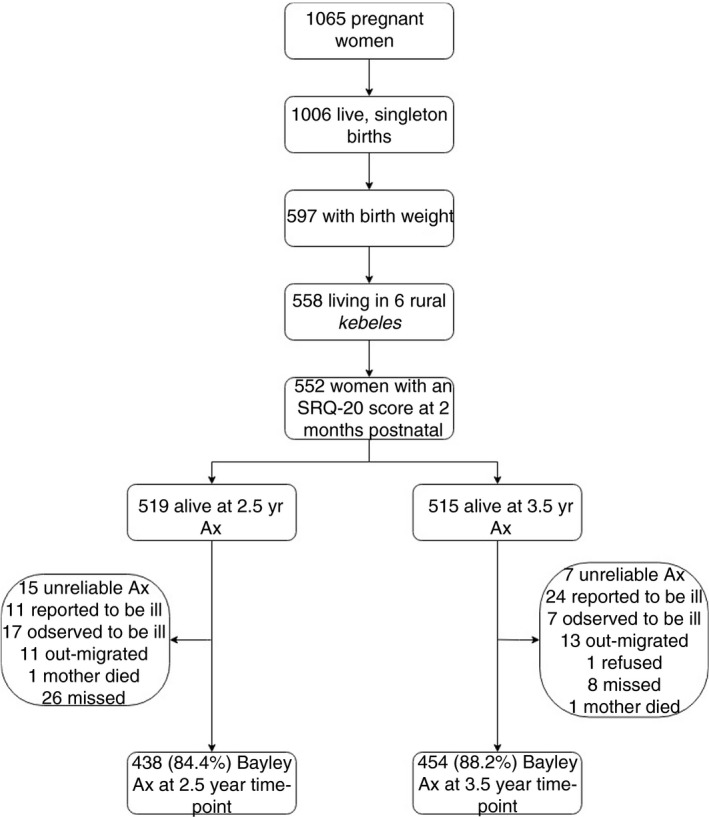
Flow diagram of follow‐up for the cohort between pregnancy, 2 months, 2.5‐ and 3.5‐year postnatal time points (SRQ‐20: Self‐reporting Questionnaire; Ax: Assessment)

A flow chart of children followed up to 2.5 and 3.5 years is presented in Figure 2. From baseline, 89.9% (496) of mother‐child dyads had complete data on the primary outcome at either 2.5 or 3.5 years. Women lost to follow‐up did not differ in SRQ‐20 score or baseline characteristics (Table S1). Those lost to follow‐up had children with lower mean birth weights than participants with data at 3.5 years (*p* = 0.04).

### Descriptive characteristics

Table 1 describes the characteristics of participating women and children. The mean maternal age at recruitment was 27.2 years (SD: 6.3). The largest ethnic group was Meskan (44.2%; *n* = 219), followed by Mareko and Silti. Most women were Muslim (80.4%; *n* = 399), and nearly all were married (99.6%; *n* = 494). A minority (2.6%; *n* = 13) reported experiencing violence at baseline. Only 10.7% (*n* = 53) of mothers had received a formal education, compared with 59.4% (*n* = 294) of fathers. The prevalence of stunting in children was 327 (74.8%) at 2.5 years and 344 (76.1%) at 3.5 years.

**TABLE 1:** Descriptive characteristics of study women and children*: at 2.5‐ (*n* = 438) and 3.5‐year (*n* = 454) time points

Characteristic	Assessed at either time point (*n* = 496)		2.5 years (*n* = 438)	3.5 years (*n* = 454)
		*n* (%)	*n* (%)	*n* (%)
Time‐fixed variables				
Ethnicity	Meskan	219 (44.2)	187 (42.7)	198 (43.6)
	Mareko	94 (19.0)	83 (19.0)	88 (19.4)
	Silti	130 (26.2)	117 (26.7)	120 (26.4)
	Other	53 (10.7)	51 (11.6)	48 (10.6)
Religious affiliation	Orthodox Christian	49 (9.9)	46 (10.5)	45 (9.9)
	Muslim	399 (80.4)	348 (79.5)	369 (81.3)
	Catholic	9 (1.8)	9 (2.1)	7 (1.5)
	Protestant	39 (7.9)	35 (8.0)	33 (7.3)
Marital status	Married	494 (99.6)	436 (99.5)	452 (99.6)
Parent's education	Neither formal education	188 (38.0)	162 (37.1)	166 (36.6)
	Either formal education	267 (53.9)	236 (54.0)	248 (54.8)
	Both formal education	40 (8.1)	39 (8.9)	39 (8.9)
Husband's age (years)	Mean (SD*)	36.5 (8.7)	36.3 (8.8)	36.6 (8.8)
Assets (0–12)	Mean (SD)	4.6 (1.4)	4.7 (1.4)	4.66 (1.4)
Parity (at baseline)	Nulliparous	59 (11.9)	53 (12.1)	53 (11.5)
	1 to 4 previous live births	274 (55.2)	242 (55.3)	249 (54.9)
	Five or more previous live births	163 (32.9)	143 (32.7)	153 (33.7)
Sex of the child	Girl	252 (50.8)	223 (50.9)	228 (50.2)
Birth weight (kg)	Mean (SD)	3.0 (0.4)	3.0 (0.4)	3.0 (0.4)
Obstetric complications	No obstetric complications	158 (33.0)	141 (33.3)	144 (33.0)
	1 obstetric complication	179 (37.4)	159 (37.6)	166 (38.0)
	≥2 obstetric complications	142 (29.7)	123 (29.1)	127 (29.1)
Marital discord scale (0–4)	Median (25^th^, 75th)	0 (0, 1)	0 (0, 1)	0 (0, 1)
Exposure to violence	Yes	13 (2.6)	12 (2.7)	13 (2.9)
Maternal age	Mean (SD)	27.2 (6.3)	27.2 (6.3)	27.3 (6.3)
Socioeconomic status (0–4)	Mean (SD)	1.4 (1.0)	1.3 (1.1)	1.4 (1.1)
Social support	No help with housework No help with children	298 (60.1) 311 (64.5)	259 (59.1) 271 (63.6)	271 (59.7) 286 (64.6)
Time‐varying variables measured at 2.5 and 3.5 years				
HOME scale^a^	Acceptance (0–5): Median (25^th^, 75th) Responsivity (0–8): Mean (SD)		5 (5, 5)	5 (5, 5)
			7.3 (1.3)	9.24 (1.5)
Infant illness episodes	Diarrhoea		237 (54.1)	137 (30.5)
	Fever		261 (59.6)	223 (49.7)
	Severe illness		212 (62.9)	112 (24.9)
Stunting	Not stunted		110 (25.2)	108 (23.9)
	Stunted (<2 Z‐scores below median)		327 (74.8)	344 (76.1)

Note

With singleton births, birth weight measured, living in rural kebeles, with an SRQ‐20 score measured 2 months postnatal.

*Standard deviation.

^a^ Home Observation for Measurement of Environment scale.

The median score (25th, 75th percentiles) on the SRQ‐20 measured at 2 months postnatal was 1 (0, 2) for mothers whose children had a Bayley III assessment at either time point, indicating low levels of CMD symptoms. The mean total Bayley development scores (SD) were 223.24 (11.29) at 2.5 years and 255.85 (8.82) at 3.5 years.

### Hypothesis‐driven analysis

In the multivariable analyses (Table 2), there was no evidence to suggest that a unit increase in maternal postnatal CMD was associated with a decrease of child development scores at 2.5 years (total scores (β^: 0.05; 95% CI: −0.25 to 0.35) or cognitive sub‐scales (β^: 0.01; 95% CI: −0.1 to 0.08). The child development scores increased by 31.72 points on the Bayley III between 2.5 and 3.5 years of the child's age (95% CI: −0.25 to 0.35) and that change did not differ according to an increase in maternal CMD score (95% CI: −0.67 to 0.4) (Table 2). A unit increase in a child's Height‐for‐Age Z‐score was associated with improved child development scores at 2.5 years by 2.06 (95% CI: 1.46 to 2.66; *p *< 0.001). An increased HOME responsivity score (β^: 0.82; 95% CI: 0.35 to 1.30; *p* = 0.01) was associated with favourable child development scores at 2.5 years, but the HOME acceptance scores were not associated (β^: 0.2; 95% CI: −0.4 to 0.81). The final model did not include exposure to violence because of lack of power. The mediating role of the home environment or stunting was not explored because of the lack of evidence for an association between maternal CMD and child development.

**TABLE 2:** Mixed‐effects model of the association between maternal postnatal common mental disorder symptoms (CMD: SRQ‐20 score) and scores on Bayley III sub‐scales at 2.5 years (main effects model), and the difference in scores on the Bayley III sub‐scales between 2.5‐ and 3.5‐year time points (interaction term with time) (*n* = 496)

Maternal CMD	Main effects model		Model with Interaction (Maternal CMD Time)		
	Total child development score	Cognitive development score	*n*	Total child development score	Cognitive development score
	β ^ (95% CI)	β ^ (95% CI)		β^ (95% CI)	β^ (95% CI)
Unadjusted model (*n* = 496)	0.05 (−0.23 to 0.32)	−0.01 (−0.1 to 0.07)	496	−0.21 (−0.73 to 0.3)	−0.13 (−0.06 to 0.17)
Model 1 (*n* = 487)	0.1 (−0.17 to 0.38)	0.001 (−0.08 to 0.09)	447	−0.18 (−0.7 to 0.34)	−0.12 (−0.28 to 0.03)
Model 2 (*n* = 480)	0.1 (−0.18 to 0.39)	−0.004 (−0.09 to 0.8)	434	−0.29 (−0.8 to 0.23)	−0.15 (−0.30 to 0.001)
Final Model (*n* = 463)	0.05 (−0.25 to 0.35)	0.01 (−0.1 to 0.08)	402	−0.14 (−0.67 to 0.4)	−0.1 (−0.26 to 0.06)

Note

Main effects model: estimated coefficients model the increase in child development score at 2.5 years for every unit increase in maternal CMD score at 2 months.

Interaction with time: estimated coefficients model the difference in the change in child development score between 2.5 and 3.5 years for every unit increase in maternal CMD score.

Model 1: Adjusted for sociodemographic characteristics: maternal age, parental education, socioeconomic status, assets, and parity.

Model 2: Adjusted for model 1 and environmental characteristics: marital discord, and social support.

Final Model: Adjusted for model 1, 2, and maternal and child characteristics: sex of the child, birth weight, child illness, and obstetric complications.

We found no evidence that the effect of postnatal CMD symptoms on child development scores varied across the levels of SES, sex of the child, social support, or stunting (Table 3).

**TABLE 3:** Adjusted estimates^a^ for the association between maternal postnatal CMD symptoms and child development stratified by gender of the child, socioeconomic status, and social support (*n* = 463)

Maternal CMD^b^		Main Effects Model				Model with Interaction (Maternal CMD × Time)			
		Total development score		Cognitive development score		Total development score		Cognitive development score	
		β^ (95% CI)	p*	β^ (95% CI)	p*	β^ (95% CI)	p*	β^ (95% CI)	p*
Stratified by									
Sex	Boys	0.22 (−0.16 to 0.59)	0.11	0.02 (−0.09 to 0.14)	0.12	−0.06 (−0.73 to 0.6)	0.66	−0.07 (−0.26 to 0.13)	0.74
	Girls	−0.31 (−0.8 to 0.19)		−0.08 (−0.23 to 0.07)		−0.14 (−0.89 to 0.6)		−0.13 (−0.4 to 0.13)	
Socioeconomic status (0–4)	Low (0–1)	0.35 (−0.17 to 0.87)	0.2	0.08 (−0.09 to 0.24)	0.21	−0.26 (−1.24 to 0.71)	0.85	−0.21 (−0.49 to 0.08)	0.46
	Average to high (2–4)	−0.05 (−0.42 to 0.32)		−0.05 (−0.16 to 0.06)		−0.19 (−0.86 to 0.48)		−0.08 (−0.28 to 0.11)	
Social support: help with housework and/or children	No	−0.17 (−0.81 to 0.46)	0.41	−0.09 (−0.28 to 0.1)	0.29	−0.95 (−2.18 to 0.27)	0.15	−0.27 (−0.59 to 0.05)	0.43
	Yes	0.15 (−0.2 to 0.49)		0.02 (−0.09 to 0.13)		0.08 (−0.5 to 0.65)		−0.07 (−0.25 to 0.11)	
Height‐for‐age Z‐score	Stunted (< −2)	0.14 (−0.18 to 0.47)	0.34	−0.005 (−0.11 to 0.1)	0.64	−0.17 (−0.78 to 0.44)	0.96	−0.09 (−0.27 to 0.09)	0.74
	Healthy (≥ −2)	−0.17 (−0.82 to 0.47)		−0.2 (−0.19 to 0.16)		0.06 (−1.06 to 1.17)		−0.12 (−0.42 to 0.19)	

Note

Main effects model: estimated coefficients model the increase in child development score at 2.5 years for every unit increase in maternal CMD score at 2 months.

Interaction with time: estimated coefficients model the difference in the change in child development score between 2.5 and 3.5 years for every unit increase in maternal CMD score.

^*^p‐value based on likelihood ratio test testing for interaction.

^a^ Adjusted for all potential confounders as described in methods.

^b^ Maternal postnatal common mental disorder symptoms (CMD: SRQ‐20 score).

### Exploratory analyses

After adjusting for all potential confounders from the hypothesis‐driven analysis, there was weak evidence of an association between maternal CMD symptoms at three years and total child development scores at 3.5 years (β^: 0.33; 95% CI: −0.04 to 0.71; *p* = 0.08) (Table S2). Only 25 (5.0%) women reported high postnatal CMD symptoms (SRQ‐20 score of six or more). There was no evidence of a threshold effect of high postnatal CMD symptoms (cut‐off of six) and total Bayley score at 2.5 years (β^: 0.77; 95% CI: −2.43 to 3.96; *p* = 0.63).

## DISCUSSION

There was no support for an independent association between postnatal CMD symptoms and overall child development or cognitive sub‐scale scores at 2.5 and 3.5 years in this population‐based cohort study from rural Ethiopia. There was no evidence for a threshold effect of CMD (≥ six on the SRQ‐20) on child development or effect modification by socioeconomic status, the sex of the child, social support, or stunting. The prospective association between maternal CMD at three years and child development at 3.5 years was close to significance, but the coefficient was small in magnitude.

The study results contrast with previous research findings. An established evidence base exists in HIC for the adverse effects of postnatal CMD on child development [49]. In LMIC, the evidence is more inconsistent, and associations have been found in urban areas where access to health care and education and SES are higher [2, 9]. Our results are similar to findings from rural regions of LMIC [12, 21, 50], enhancing their generalisability to food insecure and low‐resource settings. Findings from a multi‐country study of community‐based cohort studies in four LMIC demonstrate the heterogeneity of results [21]. For the cohorts in India and Vietnam, maternal CMD was associated with lower cognitive development, but not in Ethiopia and Peru.

In comparison, in a cross‐sectional study in Ethiopia with non‐validated maternal CMD measures, a relationship was shown between maternal depression and adverse child development [20]. In a hospital‐based study, researchers in India found that infants of depressed mothers scored lower on mental and motor scales [26]. In a community‐based quasi‐experimental study in Pakistan [43], a relationship was observed between postnatal depression and impaired child development. These results suggest cultural and regional differences specific to the Asian continent or the contextual differences in poverty rates between sub‐Saharan African and South Asian study settings [51].

Methodological differences render direct comparisons across studies in LMIC challenging, including the use of various study designs, measurement tools, choice of confounders, and assessment time points [19]. Multiple measures exist to assess early child development, but development is also determined by contextual and cultural factors that differ globally [52]. Researchers in India derived a mental and a motor quotient from the Bayley scale [26]. South African researchers assessed only the motor and cognitive subscales [23]. Formal validation of the depression and development measures was inconsistent [4, 52].

Our consistent null findings suggest that cumulative macro‐level influences, such as the financial, healthcare, and nutritional resources, are more potent influences on the adverse child development outcomes observed than maternal CMD [53]. As countries become more food secure and observe decreases in population food insecurity and stunting, one may observe more variability in child development due to individual‐level factors such as maternal CMD. Chronic protein‐energy malnutrition, which causes stunting and wasting, has been associated with the brain's structural and functional pathology, leading to adverse child development and cognitive impairment [54, 55]. Ethiopia remains a low‐income country with lower nutritional diversity and higher stunting and poverty rates than neighbouring East African countries [56, 57].

The low prevalence of maternal CMD found in this study may be explained by environmental and sociocultural protections that heighten the resilience to mental disorders in LMIC compared with HIC. Researchers have suggested that the lower prevalence of mental disorders in LMIC can be explained by the strength of social support networks, the narrower income inequality, and the lower emphasis on individual achievement [58]. Social and cultural rituals and practices around the time of birth may also protect pregnant women in this area from developing more moderate to severe symptoms of CMD in the perinatal period [35], protecting children from postnatal CMD’s adverse effects. After birth, shared parenting practices within the community may have provided a substituted caregiving relationship that protected the child [59]. In addition, most women in the study breastfed, promoting the mother‐child attachment and buffering against maternal CMD’s adverse impacts [60].

The lack of evidence for effect modification by sex of the child was inconsistent with results from HIC [17, 49, 61]. In HIC studies, boys with depressed mothers performed poorly compared with girls on development scales. The relationship between maternal CMD and child development is also established in cultures where gender biases exist, in settings with stronger preferences for male children [62].

### Limitations and strengths

Some limitations need to be addressed. Data collection took place between 2005 and 2006, potentially limiting the generalisability of results. Rates of childhood stunting under age five in Ethiopia have decreased since 2005 (46.5%) [63] but remain high (38.4%) [64]. Thus, limited financial and food security changes in this setting have occurred, and the study results remain relevant [65].

Despite including a number of *a priori* confounders, there is potential for residual confounding from unmeasured factors that affected both women's CMD and a child's development. There was no adjustment for the number of mothers who gave birth to an additional child between the two development outcome time points, which may have affected the relationship between maternal CMD and the oldest child's development. The model also did not include exposure to violence because less than 3% of participants experienced violence. In a multi‐country study, the prevalence of domestic violence in Ethiopia was estimated higher (71%) [66] than in this current study (2.6%). Exposure to violence has reported associations with maternal depression [22] and child development [25]. The prevalence of domestic violence is frequently unreported because of personal and societal reasons, including stigma, fear of retaliation, economic dependency, victim‐blaming, and societal power dynamics in many cultures [67].

The measures included in this study have some limitations. The SRQ‐20 functions as a non‐specific measure of depression and anxiety. However, the SRQ‐20 demonstrated high criterion and convergent validity in this setting, compared with other self‐report measures for maternal CMD [40]. The measure has consistently shown broader applicability cross‐culturally than other measures due to its reliability, validity, and inclusion of somatic symptoms [68]. Population‐based studies likely detect lower and more representative CMD scores, while facility‐based studies are biased toward more severe and chronic maternal CMD cases [69]. Evidence suggests a more substantial effect on child outcomes for diagnosed mental illnesses than mild symptomatology [11]. In addition, the HOME measure of environmental stimulation was not formally validated for the setting. Because of difficulties with the contextual adaptation of the HOME, we relied on the sub‐scales based on observations of mother‐child interactions.

The study does have numerous strengths, including its longitudinal design and adequate power. The multitude of child development outcome time points allowed for ascertaining temporal relationships. Given that this rural area has low health service engagement by Ethiopian women, the population‐based design facilitated recruiting a representative sample of women and children, improving the generalisability of results. Another strength of the study is that the Bayley III was contextualised for the setting and directly assessed child performance through observation, thus reducing the potential for negative recall bias [33].

### Research and policy implications

The results from this study and previous studies emphasise a need for evidence to understand the mechanisms that connect maternal CMD and child development to identify targets for intervention. WHO has recommended integrating mental health care into existing strategies to detect mothers’ and children's physical and psychological health in LMIC [70]. Evidence exists that parenting interventions [71, 72] and community‐based strategies involving the entire family are feasible and improve postnatal CMD and child development [73].

## CONCLUSION

The study's results do not support the hypothesis that postnatal CMD adversely affected child development beyond the first years of life in a rural area in Ethiopia. As countries observe decreases in population food insecurity and stunting, more variability in child development resulting from individual‐level factors, such as maternal CMD, may emerge.

## Supporting information

Supplementary Material
