# Supplementary material for: Common perinatal mental disorders and post‐infancy child development in rural Ethiopia: A population‐based cohort study
Source: Trop Med Int Health. 2022 Feb 8;27(3):251–61. doi: 10.1111/tmi.13725 (PMC9305759; doi:10.1111/tmi.13725)
Supplement: Supplementary file 1 — Supplementary Material [file TMI-27-251-s001.docx]

**Supplementary File 1** Measurement time points of the outcome, exposure, and potential confounding variables (*postnatal; CMD: common mental disorder; HOME: Home Observation for Measurement of Environment scale).


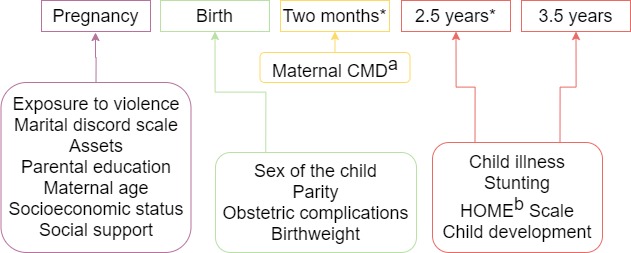


**Supplementary File 2** A comparison of baseline non-time-varying socio-demographic characteristics in participants at baseline (n=552) and excluded (lost to follow-up or with missing data on the primary outcome at 2.5 and 3.5 years).

| Characteristic |  | Baseline: (n=552) | Lost to follow up 2.5 years (n=114) | OR* comparing lost to follow up to baseline | p-value | Lost to follow up 3.5 years (n=98) | OR* comparing lost to follow up to baseline | p-value |
| --- | --- | --- | --- | --- | --- | --- | --- | --- |
|  |  | n (%) | n (%) |  |  | n (%) |  |  |
| Maternal age in years (pregnancy) | Mean (SD*) | 27.2 (6.3) | 27.1 (6.5) |  | 0.91^a^ | 26.5 (6.3) |  | 0.26^a^ |
| Ethnicity | Meskan | 237 (42.9) | 50 (43.9) | Ref | 0.85^b^ | 39 (39.8) | Ref | 0.83^b^ |
|  | Mareko | 106 (19.2) | 23 (20.2) | 1.04 (0.59-1.81) |  | 18 (18.4) | 1.04 (0.56-1.92) |  |
|  | Silti | 148 (26.8) | 31 (27.2) | 0.99 (0.59-1.64) |  | 28 (28.6) | 1.18 (0.69-2.03) |  |
|  | Other | 61 (11.1) | 10 (8.8) | 0.73 (0.35-1.55) |  | 13 (13.3) | 1.38 (0.68-2.78) |  |
| Religious affiliation | Orthodox Christian | 54 (9.8) | 8 (7.0) | Ref | 0.26^c^ | 9 (9.2) | Ref | 0.83^c^ |
|  | Muslim | 447 (81.0) | 99 (86.8) | 1.64 (0.75-3.59) |  | 78 (79.6) | 1.06 (0.50-2.25) |  |
|  | Catholic | 9 (1.6) | 0 (0.0) | 0.00 |  | 2 (2.0) | 1.43 (0.25-8.16) |  |
|  | Protestant | 42 (7.6) | 7 (6.1) | 1.15 (0.38-3.50) |  | 9 (9.2) | 1.36 (0.48-3.84) |  |
| Marital status | Married | 549 (99.5) | 113 (99.1) | 0.52 (0.04-5.78) | 0.50^c^ | 97 (99.0) | 0.43 (0.04-4.80) | 0.44^c^ |
| Parent’s education | Both formal education | 49 (8.9) | 10 (8.85) | Ref | 0.37^d^ | 10 (10.3) | Ref | 0.07^d^ |
|  | Either formal education | 289 (52.6) | 53 (46.9) | 0.90 (0.41-1.87) |  | 41 (42.3) | 0.64 (0.30-1.40) |  |
|  | Neither formal education | 212 (38.6) | 50 (44.25) | 1.20 (0.56-2.59) |  | 46 (47.4) | 1.1 (0.50-2.33) |  |
| Socioeconomic Status | Mean (SD*) | 1.4 (1) | 1.4 (1) |  | 0.69^a^ | 1.3 (1) |  | 0.69^a^ |
| Assets (0-12) | Mean (SD) | 4.6 (1.4) | 4.4 (1.2) |  | 0.08^a^ | 4.5 (1.4) |  | 0.2^a^ |
| Parity (at baseline) | Nulliparous | 70 (12.7) | 17 (14.9) | Ref | 0.79^d^ | 18 (18.4) | Ref | 0.88^d^ |
|  | 1 to 4 previous live births | 305 (55.3) | 63 (55.3) | 0.81 (0.44-1.50) |  | 56 (57.1) | 0.65 (0.35-1.19) |  |
|  | Five or more previous live births | 177 (32.1) | 34 (29.8) | 0.74 (0.38-1.44) |  | 24 (24.5) | 0.45 (0.23-0.90) |  |
| Infant gender | Girl | 277 (50.2) | 54 (47.4) | 0.87 (0.57-1.31) | 0.5^b^ | 49 (50.0) | 0.99 (0.64-1.53) | 0.97^b^ |
| Birth weight (kg) | Mean (SD) | 3.0 (0.4) | 3.0 (0.4) |  | 0.29^a^ | 2.9 (0.4) |  | 0.04^a^ |
| Obstetric complications | No obstetric complications | 176 (33.0) | 35 (31.8) | Ref | 0.26^d^ | 32 (33.3) | Ref | 0.1^d^ |
|  | 1 obstetric complication | 194 (36.4) | 35 (31.8) | 0.87 (0.53-1.50) |  | 28 (29.2) | 0.76 (0.44-1.32) |  |
|  | ≥2 obstetric complications | 163 (30.6) | 40 (36.4) | 1.31 (0.78-2.20) |  | 36 (37.5) | 1.28 (0.75-2.17) |  |
| ^a^ p-value based on t-test  ^b^ p-value based on Pearson’s χ²-test  ^c^ p-value based on Fisher’s exact test | | | | ^d^ p-value based on likelihood ratio test assessing for trend  ^e^ p-value based on Wilcoxon rank-sum test  * SD: standard deviation & OR: odds ratio | | | | |

**Supplementary File 3** Linear regression model of the association between maternal postnatal common mental disorder symptoms (CMD: SRQ-20 score) at three years and scores on Bayley III sub-scales in 3.5-year-old children (n=452).*

| Maternal CMD | Total child development score | | Cognitive development score | |
| --- | --- | --- | --- | --- |
|  | β^ | 95% CI | β^ | 95% CI |
| Unadjusted β^ (n=496) | 0.3 | -0.06 to 0.65 | 0.02 | -0.09 to 0.13 |
| Model 1 (n=447) | 0.33 | -0.02 to 0.69 | 0.03 | -0.07 to 0.14 |
| Model 2 (n=434) | 0.31 | -0.05 to 0.68 | 0.03 | -0.09 to 0.14 |
| Final Model (n=402) | 0.33 | -0.04 to 0.71 | 0.04 | -0.08 to 0.16 |
| * In the analytical sample of participants with singleton births, birth weight measured, living in rural kebeles, with an SRQ-20 score measured two months postnatal.  Model 1: Adjusted for socio-demographic characteristics: maternal age, parental education, socioeconomic status, assets, and parity.  Model 2: Adjusted for model 1 & environmental characteristics: marital discord, and social support  Final Model: Adjusted for models 1, 2, and maternal & child characteristics: sex of the child, birth weight, child illness, and obstetric complications. | | | | |
